# The increased effective connectivity from left middle occipital gyrus to right medial septum/diagonal bands in AD patients after donepezil intervention

**DOI:** 10.3389/fnagi.2024.1362790

**Published:** 2024-04-10

**Authors:** Ting Yang, Fuquan Wei, Yufei Guo, Mengxiao Zhu, Hongtao Hou, Zhongwei Guo, Xiaozheng Liu

**Affiliations:** ^1^The Second Affiliated Hospital and Yuying Children’s Hospital, Wenzhou Medical University, Wenzhou, Zhejiang, China; ^2^Tongde Hospital of Zhejiang Province, Hangzhou, Zhejiang, China; ^3^The Second Clinical Medical College, Zhejiang Chinese Medical University, Hangzhou, Zhejiang, China; ^4^Wenzhou Key Laboratory of Structural and Functional Imaging, Wenzhou, Zhejiang, China

**Keywords:** Alzheimer’s disease, cholinergic, functional magnetic resonance imaging, donepezil, effective connectivity

## Abstract

**Introduction:**

Donepezil enhances the function of cholinergic nerves by increasing the concentration of acetylcholine, thereby improving clinical symptoms in patients with Alzheimer’s disease (AD). However, the neural mechanisms of how donepezil modulates the effective connectivity (EC) network of cholinergic system in AD patients remain unknown. We speculated that the effective network of the cholinergic system changes in AD patients after donepezil intervention.

**Methods:**

We employed resting-state functional magnetic resonance imaging and Granger causality analysis approach to explore changes in the effective connectivity network of the basal forebrain in AD patients before and after donepezil intervention. This study included 32 participants, including 16 healthy controls (HCs) and 16 AD patients. In a 3T MRI scanner, the 16 AD patients were scanned before and after the donepezil intervention. To compare EC differences between the three groups of participants, ANOVA and *post-hoc t*-tests analysis were employed.

**Results:**

Compared to baseline status, AD patients after donepezil intervention had an increased EC from left middle occipital gyrus to right medial septum/diagonal bands. Compared to HCs, AD patients after donepezil intervention had an increased EC from right inferior frontal gyrus/orbit part to right medial septum/diagonal bands, AD patients before donepezil intervention had a reduced EC from right precuneus to right medial septum/diagonal bands. A significant positive correlation was found between EC values in right precuneus and Mini-Mental State Examination in pre-intervention AD patients (*r* = 0.7338, *p* = 0.0012).

**Discussion:**

Our study showed that effective connectivity of brain regions associated with the default mode network in the cholinergic pathway was enhanced after donepezil intervention. The results of this study will help us to better understand the neural mechanisms of donepezil intervention in AD and to find clinical targets for intervention.

## 1 Introduction

Cholinesterase inhibitors are currently the first line of treatment for AD patients in clinical practice (Renn et al., 2018). Among them, donepezil is highly selective for acetylcholinesterase in the central nervous system (CNS). Cholinesterase inhibitors increases the concentration of acetylcholine in the CNS, particularly in cortical and basal forebrain (BF) synapses, little effect on peripheral acetylcholinesterase, thus effectively improving cognition. Donepezil hydrochloride is therefore widely used in clinical practice (Fan et al., 2022). AD is considered a “disconnection syndrome,” suggesting that loss of neurons and their connections disrupts the structural and functional connections between neurons and macroscopic brain regions, leading to clinical symptoms (Stam, 2014). Therefore, understanding the brain mechanisms by which donepezil modulates the cholinergic network to intervene in the clinical symptoms of AD may help to improve the theoretical basis for clinical drug selection.

In recent years, magnetic resonance imaging (MRI) technology has been used to study the brain mechanisms of donepezil intervention in AD (Saykin et al., 2004; Dubois et al., 2015; Griffanti et al., 2016; Luo et al., 2019). Short-term treatment with cholinesterase inhibitors enhances neuronal activity of frontal circuits in mild cognitive impairment (MCI) patients, and this increase is associated with improved cognitive performance and baseline hippocampal integrity (Saykin et al., 2004). The rate of hippocampal atrophy in prodromal AD was reduced by 45% after 1 year of treatment with donepezil compared to the placebo group (Dubois et al., 2015). Studies of functional connectivity showed an increase in functional connectivity (FC) in the orbitofrontal neural network in AD patients after donepezil intervention and correlated with post-treatment cognitive improvement (Griffanti et al., 2016). However, it has been shown that there is not only strength of connection but also directionality in the correlation between brain regions (Luo et al., 2019). A cross-sectional study of the Granger causality analysis (GCA) showed reduced EC from the posterior cingulate to the middle temporal gyrus, anterior cingulate and precuneus in patients with AD carried by APOEε4 (Luo et al., 2019). However, there are no studies examining the neural mechanisms underlying the effective connectivity network of cholinergic pathways in AD patients before and after donepezil intervention.

The basal forebrain can be divided into the nucleus basalis of Meynert and the medial septum/diagonal bands (MS/DB), depending on the brain region to which the cholinergic nerves project remotely (Zaborszky et al., 1999). Castaneda et al. (2015) explored changes in cholinergic neurons in a rat model in which the medial septal nucleus was injected with Aβ1-40 and memantine. They found that the number of cholinergic neurons in the medial septum and diagonal band was significantly reduced in the Aβ1-40 group compared to the Aβ/memantine treated group (Castaneda et al., 2015). Reduced MS/DB gray matter volume in AD patients also correlates with spatial cognitive function (Parizkova et al., 2020). Selective activation of cholinergic circuits that branch vertically from the medial septal nucleus of the basal forebrain and the diagonal band nucleus to the hippocampus attenuates memory deficits in APPswe/PSEN1dE9 (APP/PS1) mice (Liu et al., 2022). However, studies investigating the mechanisms of MS/DB-related brain networks in AD patients are still scarce.

Thus, we aimed to examine EC network modifications in cholinergic pathways in Alzheimer’s disease patients following donepezil administration using rsfMRI data and the GCA approach. For EC analysis, we used the MNI standard space’s basal forebrain subregions template as the region of interest. We predicted that changes in the basal forebrain EC network are linked to cognitive function in Alzheimer’s disease patients.

## 2 Materials and methods

### 2.1 Patients

Between January 2018 and July 2022, sixteen individuals with Alzheimer’s disease participated in this longitudinal study at Tongde Hospital in Hangzhou, Zhejiang Province, China. According to the National Institute on Aging-Association Alzheimer’s recommendations (Gmitrowicz and Kucharska, 1994), patients with AD matched the criteria for probable AD. They were right-handed, had a CDR of 0.5, a Mini-Mental State Examination (MMSE) score of less than 24, and had completed more than six years of education. Sixteen healthy people were enrolled to serve as healthy controls (HC). They were cognitively normal and had a CDR score of 0. Participants with a history of mental illness, who were using antidepressants, or who had MR imaging contraindications were eliminated.

To determine the level of depression, the neuropsychiatric inventory (NPI) (Gmitrowicz and Kucharska, 1994) and the Cornell scale for depression in dementia (CSDD) (Alexopoulos et al., 1988) were employed. Depression symptoms were declared present when the CSDD score was larger than 6 and the NPI depression domain score was larger than 4 (Alexopoulos et al., 1988; Cummings et al., 1994; Schneider et al., 2001).

At baseline (T1) and after 24 weeks (T2) of donepezil treatment, patients had MRI scans, neurological and medical examinations, and neuropsychological assessments (5 mg daily for the first 4 weeks, then 10 mg daily) (Zhang and Gordon, 2018). Everyone who took part signed an informed consent form. The Ethics Committee approved the study.

### 2.2 MRI scanning

This study was performed using a Siemens Magnetom Verio (Siemens Medical Systems, Erlangen, Germany), a 3.0 Tesla MRI scanner with an 8-channel head coil. Fast gradient echo images of anatomical T1-weighted whole brain magnetization-prepared were obtained using the following parameters: TI/TR/TE = 900/1,900/2.48 ms, 128 slices, 1 mm thickness, 0 mm gap, 9° FA, 256 × 256 acquisition matrix, 256 mm × 256 mm FOV. Gradient echo planar imaging was used to provide axial functional images with the following parameters Images: repetition rate (TR) = 2,000 ms, echo time (TE) = 30 ms, slice = 33, thickness = 4.8 mm, gap = 0 mm, field of view (FOV) = 200 mm × 200 mm, acquisition matrix = 64 × 64, flip angle = 90°. It took 6 min 40 s to complete the fMRI scan.

### 2.3 Data processing

Data preprocessing was done using SPM12^1^ and Resting-State fMRI Data Analysis Toolkit plus V1.25 (RESTplus V1.25).^2^ The first ten volumes were eliminated. Slice-timing was carried out and motion correction was applied. In terms of framewise displacement, participant head motion was minimal (mean FD < 0.5). We first coregistered the fMRI images to each subject’s high-resolution T1 anatomical scan before normalizing them to Montreal Neurological Institute (MNI) space. The images were then adjusted to match the MNI152 template. 6 mm full widths at half maximum Gaussian kernel were used to smooth the normalized images and linear detrending. To reduce low-frequency drift and physiological high-frequency respiration, temporal bandpass filtering (0.01–0.08 Hz) were applied. White matter, cerebrospinal fluid and head motion artifacts were regressed out to eliminate false signals.

### 2.4 Effective connectivity calculation

**FIGURE 1:**
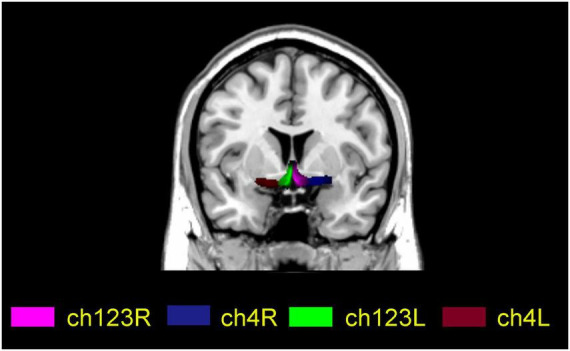
Localization map of basal forebrain subregions on a standard MNI spatial template. Ch123L and Ch123R indicate the left and right medial septum/diagonal bands, respectively; Ch4L and Ch4R indicate the left and right basal nuclei of Meynert, respectively.

For each participant, four BF seeds (two per hemisphere) were created using a method based on Julich-Brain Atlas (Schneider et al., 2001), respectively. The four subregions are ch123 in the left and right medial septum/diagonal bands and Ch4 in the left and right basal nuclei of Meynert (Figure 1).

Each subject underwent a voxel-wise GCA analysis using RESTplus V1.25. Four BF subregions were chosen for each participant in accordance with the available literature (Amunts et al., 2020). The seed time series X is the average time series of the seed regions, and the time series Y is the time series of the remaining voxels across the entire brain. In the entire brain, the linear direct effects of X on Y (information flow from X to Y) and Y on X (information flow from Y to X) are assessed voxel by voxel. In terms of directionality, a positive coefficient from X to Y denotes a causal relationship between activity in area X and Y. A negative coefficient from X to Y shows that activity in area X has a direct opposite influence on activity in area Y. A GCA map from the BF subregions to the whole brain (x2y) and a GCA map from the whole brain to the BF subregions (y2x) are represented as the results for each subject. For second-level group analyses, each individual-level EC map was then transformed using Fisher’s r-to-z transformation into a z-map (Zang et al., 2012).

### 2.5 Statistical analysis

The demographics and clinical characteristics were analyzed using statistical software (Statistical Package for the Social Sciences v.15.0; SPSS, Inc., Chicago, IL, USA). A two sample *t*-test was used to analyze differences in age and education, and chi-squared tests were used to analyze differences in gender distribution.

Prior to statistical analyses, we tested the normality of the functional MRI data using the Lilliefors test. A one-way analysis of covariance (ANCOVA) was used to compare the BF EC maps voxel by voxel between the three groups. Then, from the ANCOVA analysis, we extracted brain masks that showed significant differences. Finally, we performed *post-hoc t*-tests between each pair of groups using the ANCOVA brain masks. The two sample *t*-test was used to compare the AD group to the HC group after the intervention, and the paired *t*-test was used to compare the AD group before and after the intervention. To ensure the accuracy of the results, we removed the mean relative displacements of head motion, age and gender as covariates in the ANCOVA and *t*-tests. The resulting statistical map was set to *p* < 0.05 for multiple comparisons (AlphaSim corrected for multiple comparisons, with a combined individual voxel *p*-value 0.005 with a cluster size > 25 voxels).

### 2.6 Relationship of EC with clinical variables

The mean BF EC values of the abnormal brain regions were extracted, and Pearson correlations were used to investigate the relationships between abnormal FC values and clinical variables in AD patients before and after intervention (*P* < 0.05).

## 3 Results

### 3.1 Neuropsychological results

Table 1 displays the demographics and clinical data. Age, gender distribution, and educational attainment differences between groups were not statistically significant (*p* = 0.107, 0.157, and 0.240, respectively). The AD group had significantly lower MMSE scores and higher NPI scores than the HC group (*p* < 0.001).

**TABLE 1:** Demographics and neuropsychological data.

	AD group	HC group	c2/*t*-value	*P*-value
Gender, *n* (M/F)	16 (8/8)	16 (7/9)	2	0.157
Age, years	65.2 ± 8.1	69.1 ± 4.5	1.659	0.107
Education, years	9.1 ± 2.0	8.3 ± 2.1	1.198	0.240
MMSE	19.7 ± 2.6	29.1 ± 0.90	−10.342	< 0.001
MMSE (24w)	20.1 ± 2.5			
NPI	4.56 ± 2.9	0.15 ± 0.36	12.632	< 0.001
NPI (24w)	1.38 ± 1.2			
CSDD	3.25 ± 2.40			
CSDD (24w)	0.75 ± 0.71			

Data are represented as the mean ± SD. The Chi-squared test was used to compare sex, and two-sample *t*-tests were used to compare age and neuropsychological data. HCs, healthy controls; AD, Alzheimer’s disease; M, male; F, female; MMSE, Mini-Mental State Examination; NPI, the neuropsychiatric inventory; CSDD, Cornell scale for depression in dementia. CDR, clinical dementia rating.

### 3.2 Abnormal BF EC values in the AD group

**FIGURE 2:**
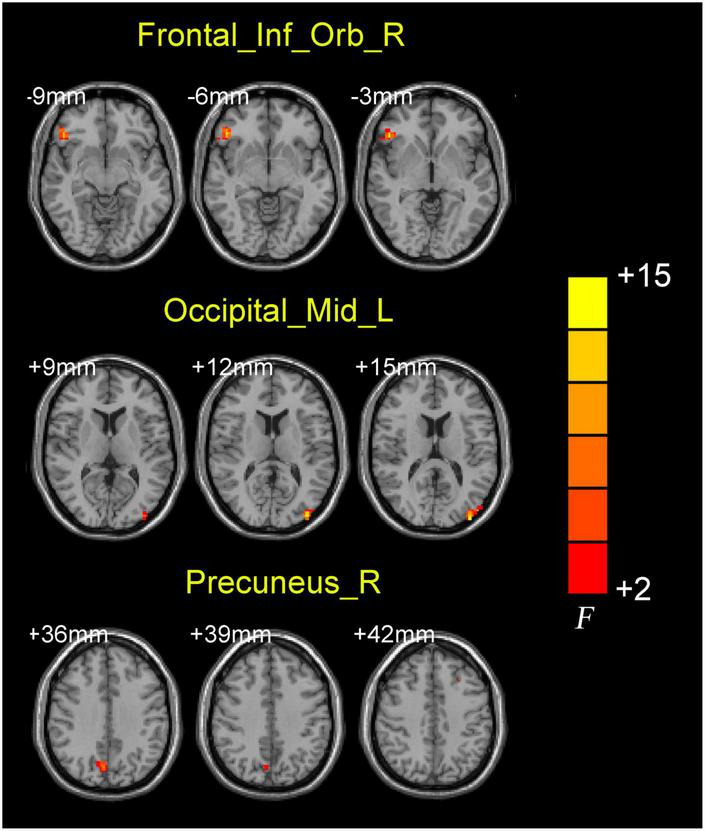
Brain regions showing the abnormal right medial septum/diagonal bands functional connectivity values among three groups.

**FIGURE 3:**
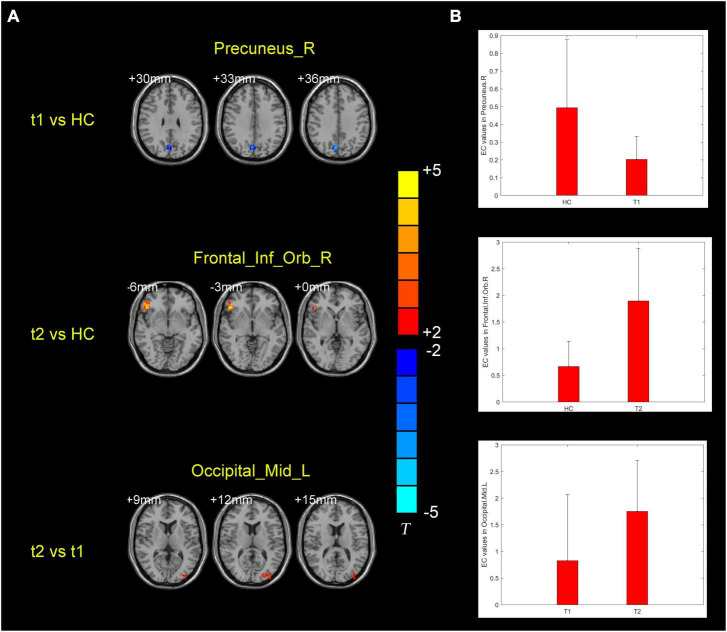
**(A)** Brain regions showing the alternation of right medial septum/diagonal bands effective connectivity values in the post-intervention AD group compared with pre-intervention AD and HC groups. **(B)** Means and standard deviations of the difference brain regions obtained from the comparison of the three groups of effective connectivity. t1, pre-intervention AD patients; t2, post-intervention AD patients.

ANCOVA analysis revealed significant EC differences between the three groups in brain regions located in the left middle occipital gyrus, right inferior frontal gyrus/orbit part and right precuneus (Table 2 and Figure 2). Compared to baseline status, AD patients after donepezil intervention had an increased EC from left middle occipital gyrus to right MS/DB. Compared to HC, AD patients after donepezil intervention had an increased EC from right inferior frontal gyrus/orbit part to right MS/DB, AD patients before donepezil intervention had a reduced EC from right precuneus to right MS/DB (Table 2 and Figure 3).

**TABLE 2:** Brain regions with significantly different effective connectivity values with right medial septum/diagonal bands in the AD after intervention group compared with the AD group before intervention and HC group.

Brain regions	Voxels	BA	MNI coordinates			F/*t*-value	*P*-value
			**x**	**y**	**z**		
**ANCOVA**							
Frontal_Inf_Orb_R	53	47	48	36	−6	14.0965	< 0.005
Occipital_Mid_L	36	19	−39	−87	12	14.5534	< 0.005
Precuneus_R	26	7	6	−72	36	10.0772	< 0.005
**t2 vs. t1**							
Occipital_Mid_L	28	19	−39	−90	12	4.3210	< 0.005
**t1 vs. HC**							
Precuneus_R	40	7	6	−72	36	−4.3702	< 0.005
**t2 vs. HC**							
Frontal_Inf_Orb_R	74	47	48	33	−3	5.2935	< 0.005

AD, Alzheimer’s disease; MNI, Montreal Neurological Institute; BA, Brodmann area. t1, pre-intervention AD patients; t2, post-intervention AD patients.

### 3.3 Correlations of EC with clinical variables

**FIGURE 4:**
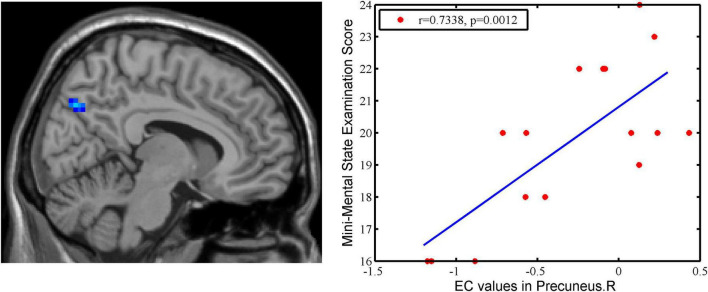
The correlation between EC values in the right precuneus and clinical variables in AD patients. There is a significant positive correlation was found between EC values in right precuneus and MMSE in pre-intervention AD patients (*r* = 0.7338, *p* = 0.0012).

A significant positive correlation was found between EC values in right precuneus and Mini-Mental State Examination in pre-intervention AD patients (*r* = 0.7338, *p* = 0.0012) (Figure 4).

## 4 Discussion

Using rsfMRI data and the GCA method, we investigated the alternation of the BF EC network in the AD groups before and after donepezil intervention in comparison to the HC group. Compared with their baseline status, AD patients after donepezil intervention had an increased EC from left middle occipital gyrus to right MS/DB. Compared to HCs, AD patients after donepezil intervention had an increased EC from right inferior frontal gyrus/orbit part to right MS/DB, AD patients before donepezil intervention had a reduced EC from right precuneus to right MS/DB. A significant positive correlation was found between EC values in right precuneus and Mini-Mental State Examination in pre-intervention AD patients.

Our research shows that AD patients after donepezil intervention exhibited an increased EC from right inferior frontal gyrus/orbit part and right precuneus to right MS/DB. The precuneus and orbitofrontal lobes belong to the default mode network (DMN). The DMN is a key brain network in the development of AD and is also the main brain network associated with cognitive function (Gomez-Ramirez and Wu, 2014). The donepezil-treated group showed increased volume of the precuneus and higher connectivity in the default mode network area compared to the untreated group (Solé-Padullés et al., 2013; Moon et al., 2016). High frequency stimulation of the precuneus in AD patients using repetitive transcranial magnetic stimulation (rTMS) was found to improve situational cognitive function in AD patients (Koch et al., 2018). Analysis of TMS-electroencephalogram (EEG) signals showed increased neural activity in the precuneus and altered functional connectivity with the medial frontal region of the DMN in AD patients (Koch et al., 2018, 2022). A Single-photon emission computed tomography (SPECT) imaging-based study showed a significant increase in preconditioning stress, disinhibition and euphoria and a significant decrease in bilateral orbital frontal perfusion in responsive AD patients compared to non-responsive AD patients after donepezil intervention (Mega et al., 2000). Our findings are consistent with those of the previous study. Donepezil did not only enhance functional, but effective connections between the basal forebrain and the default mode network, thereby improving clinical symptoms in AD patients.

Our results show that, comparison to their baseline status, AD patients after donepezil intervention had an increased EC from left middle occipital gyrus to right MS/DB. Visual dysfunction is one of the main clinical symptoms of AD patients, symptoms including spatial disturbances and reduced visual field for cognitive range of vision (Kusne et al., 2017). Better orientation performance in AD patients is associated with enhanced cerebral metabolism in the bilateral middle and inferior temporal lobes, bilateral middle and posterior cingulate gyrus, left angular gyrus and left middle occipital gyrus. Orientation functions include list learning, recognition memory, visuospatial function, attention and language (Weissberger et al., 2017). Four hours after donepezil administration, there was a significant relative increase in perfusion in the left parietal, right superior frontal gyrus and right middle occipital gyrus in responders (Tepmongkol et al., 2019). Our findings suggest that donepezil enhances the strength of connections between the basal forebrain and middle occipital gyrus, thereby improving visual cognitive function in AD patients.

Our study adds to the neural mechanisms of donepezil intervention on clinical symptoms in AD patients. Previous findings have shown that donepezil modulates clinical symptoms in AD patients from different perspectives, such as hippocampal volume, frontal functional networks and MAPK/NLRP3 inflammasome/STAT3 signaling (Dubois et al., 2015; Griffanti et al., 2016; Kim et al., 2021). Moreover, the combination of machine learning methods and imaging data can provide guidance for clinical drug use. By increasing the amount of training data and the generalization ability of the machine learning model, it can better predict the efficacy of clinical drugs for AD patients and guide the use of clinical drugs (May, 2021).

We should be aware of some restrictions on our study. To further validate the accuracy of the results, larger samples and data from multiple sites are first required. Second, a growing body of evidence suggests that cognitive changes in preclinical AD may be more global (Salmon, 2012). Thus, more comprehensive scales, like the Montreal Cognitive Assessment and the Alzheimer’s Disease Assessment Scale-cognitive subscale, could be used to assess the clinical status of AD patients since the scales used in this study to measure the cognitive function of AD patients are not sufficiently rich. Third, this study used rsfMRI to examine changes in the functional network of the cholinergic pathway in the gray matter of AD patients. Diffusion imaging may be used in the future to investigate abnormalities in the connections between white matter fibers in the cholinergic pathway in AD patients. Finally, T2-weighted MR images were not collected in this study to assess white matter hypersignal (WMH) in the patients. WMH loading indicates the severity of possible cerebral small-vessel disease or other cerebrovascular abnormalities, and may be an important influencing factor.

## 5 Conclusion

In the current study, we compared the BF EC in AD groups before and after donepezil intervention and HC groups. Our results showed that abnormal brain regions are located on the DMN and occipital lobe. These findings suggest that donepezil enhances the strength of connections between the basal forebrain with DMN and middle occipital gyrus, thereby improving cognitive function in AD patients.

## Data availability statement

The original contributions presented in this study are included in the article/supplementary material, further inquiries can be directed to the corresponding authors.

## Ethics statement

The studies involving humans were approved by the Ethics Committee of Tong De Hospital of Zhejiang Province (approval no. 2017-11-12). The studies were conducted in accordance with the local legislation and institutional requirements. The participants provided their written informed consent to participate in this study.

## Author contributions

TY: Formal analysis, Investigation, Methodology, Visualization, Writing – original draft, Writing – review & editing. FW: Investigation, Methodology, Resources, Software, Writing – review & editing. YG: Formal analysis, Resources, Data curation, Validation, Writing – review & editing. MZ: Data curation, Formal analysis, Investigation, Validation, Writing – review & editing. HH: Investigation, Methodology, Software, Writing – review & editing. ZG: Formal analysis, Conceptualization, Funding acquisition, Project administration, Resources, Writing – review & editing. XL: Software, Conceptualization, Funding acquisition, Project administration, Writing – original draft, Writing – review & editing.

## Footnotes

1
http://www.fil.ion.ucl.ac.uk/spm

2
http://www.restfmri.net
